# Fish Consumption and Risk of Rheumatoid Arthritis: Findings from the E3N Cohort Study

**DOI:** 10.3390/nu14040861

**Published:** 2022-02-18

**Authors:** Yann Nguyen, Carine Salliot, Xavier Mariette, Marie-Christine Boutron-Ruault, Raphaèle Seror

**Affiliations:** 1 Centre for Research in Epidemiology and Population Health (CESP), INSERM U1018, Université Paris-Saclay, 94807 Villejuif, France; yann.nguyen2@aphp.fr (Y.N.); carine.salliot@chr-orleans.fr (C.S.); marie-christine.boutron@gustaveroussy.fr (M.-C.B.-R.); 2 Department of Internal Medicine, AP-HP. Nord, Hôpital Beaujon, Université de Paris, 92110 Clichy, France; 3 Rheumatology Department, Centre Hospitalier Régional d’Orléans, 45067 Orléans, France; 4 Centre of Immunology of Viral Infections and Auto-Immune Diseases (IMVA), INSERM U1184, Université Paris-Sud, Université Paris-Saclay, 94270 Le Kremlin Bicêtre, France; xavier.mariette@aphp.fr; 5 Rheumatology Department, AP-HP, Hôpitaux Universitaires Paris-Saclay—Hôpital Bicêtre, 94270 Le Kremlin Bicêtre, France

**Keywords:** rheumatoid arthritis, diet, fish consumption, risk factor

## Abstract

Fish consumption has been thought to reduce the risk of rheumatoid arthritis (RA), but the reported data are conflicting. We aimed to assess the association between fish consumption (overall, lean fish, and oily fish) and the risk of RA. The E3N Study is a French prospective cohort study including 98,995 women since 1990. Dietary data were collected via a validated food frequency questionnaire in 1993. Cox proportional hazards models were used to calculate HRs and 95% CIs for incident RA. Models were adjusted for age and for the main potential confounders including cigarette smoking. Among 62,629 women, 480 incident cases of RA were identified. In the overall population, we did not find a linear association between overall fish consumption and RA risk (*p* for trend 0.65), but a moderate consumption of fish was associated with a decreased risk of RA (HR 0.74; 95% CI 0.59–0.94 for tertile 2 compared with tertile 1), especially among current or former smokers (HR 0.61; 95% CI 0.44–0.85). Although not statistically significant, a trend towards an inverse association was only found with oily fish consumption (HR 0.81; 95% CI 0.65–1.02), but not with lean fish. Our results suggest that moderate fish consumption could reduce the risk or RA and potentially counterbalance the increased risk of RA induced by smoking. This inverse association might be explained by the omega-3 fatty acid content of oily fish.

## 1. Introduction

Rheumatoid arthritis (RA) is an inflammatory rheumatoid disease, in which an interaction between genetic factors and environment could trigger autoimmunity and play a role in its pathogenesis [1]. Cigarette smoking is a major risk factor of RA, especially for seropositive RA (with positive anti-citrullinated protein antibodies (ACPA)), among genetically predisposed patients [2].

Diet has been thought to play a role in RA pathophysiology [3]. Special attention has been given to fish consumption, as fish is rich in omega-3 fatty acids. Moderate fish consumption has been thought to be associated with lower risk of cardiovascular diseases and mortality in some populations [4]. Regarding the risk of RA, increased intake of omega-3 fatty acids has been suggested to decrease the risk of developing ACPAs and to prevent the onset of RA once ACPAs have been detected [3]. Numerous studies have assessed the association between fish intake and RA, but the results have been conflicting [5,6,7,8,9]. In a case-control study, Shapiro et al. found that a high consumption of broiled or baked fish was associated with a lower risk of incident RA (odds ratio 0.57; 95% CI [0.35; 0.93]) [10]. However, this association was not found with other types of fish. Nevertheless, no linear association was found in at least four prospective cohort studies and three other case-control studies [6,9,11,12,13,14]. In a meta-analysis including 174,701 participants, Di Giuseppe et al. did not find any association between an increased intake of fish and the risk of RA (≥1 serving/week compared to <1; relative risk 0.71; 95% CI 0.48–1.04). However, case-control studies could be prone to recall bias, and separate analyses by subtypes of fish consumption (lean fish or oily fish, the latter being the main source of omega-3 fatty acids [15]) were often not available.

Thus, we aimed to further analyse the possible association between fish intake and the risk of RA in our large prospective general population cohort of French women: the E3N cohort (Etude Epidémiologique auprès des femmes de la Mutuelle générale de l’Education Nationale).

## 2. Materials and Methods

### 2.1. Study Population

The E3N study (Etude Epidémiologique auprès des femmes de la Mutuelle générale de l’Education Nationale) is a French prospective cohort study following 98,995 French women since 1990. This study was conducted to study environmental factors associated with cancers and chronic diseases. Women were born between 1925 and 1950, and were covered by a national health insurance primarily involving teachers [16]. Participants were asked to complete biennially mailed questionnaires (Q1 to Q12) assessing their health, lifestyle, and newly diagnosed diseases. Less than 3% of women were lost to follow-up since 1990, and the average response rate was 83% for each questionnaire. 

### 2.2. RA Ascertainment

Identification of RA cases has been previously described in a dedicated validation study [17]. Briefly, women who self-reported a diagnosis of RA in three follow-up questionnaires (Q9, Q10, and Q11) were sent a specific questionnaire derived from Guillemin et al. in 2017 [18]. Participants were considered as RA cases if they confirmed having RA in the specific questionnaire and any of the following: (1) if they self-reported that RA was confirmed by a rheumatologist or another physician, (2) if they self-reported being prescribed any disease-modifying antirheumatic drugs considered specific to RA, (3) if they self-reported having positive autoantibodies (rheumatoid factor and/or ACPA), or (4) if they self-reported having at least four of the seven 1987-ACR criteria. The sensitivity and specificity of this algorithm were 93.8% and 82.6%, respectively. If they did not answer the specific validation questionnaire, there were considered as RA cases if they had reimbursement of any medication considered to be specific for RA in the medication reimbursement database. The sensitivity and specificity of this method were 70.5% and 87.3%, respectively.

For the present study, only incident cases, diagnosed after Q3 (dietary questionnaire) were included, and we excluded women who did not complete any of the three questionnaires (Q9, Q10, and Q11) collecting a self-reported diagnosis of RA, prevalent RA cases (occurring before baseline), and cases without date of diagnosis.

### 2.3. Fish Consumption

At the same time as the third questionnaire (Q3), between 1993 and 1995, a specific dietary questionnaire was sent [19]. This questionnaire included qualitative questions on food groups, and quantitative questions on the intake and the frequency of food group consumption. To help the participants, a booklet of pictures of portion sizes was sent. The consumption of 208 foods was assessed. The reproducibility of this questionnaire was satisfied in a dedicated study [19].

Daily consumption of fish was estimated in gram per days and included the consumption of the lean fishes (hake, pollack, common ling, dab, haddock, sole, whiting, and/or cod) and oily fishes (sardine, mackerel, salmon, trout, and/or tuna).

Women were classified into approximate tertiles of fish consumption (low, moderate, or high consumption), lean fish consumption, and oily fish consumption.

### 2.4. Other Variables

Data on education level (<high school, up to two-level university, or two-level university or more), personal cigarette smoking (current smoker, former smoker, or non-smoker), exposure to passive smoking in childhood, body mass indexes (BMI, in kg/m^2^), gastrointestinal disorders (normal, diarrhoea, constipation, or alternating diarrhoea/constipation), and history of *diabetes mellitus* or hypothyroidism were collected during baseline.

Other dietary data, including the consumption of fruits, vegetables, olive oil, cereal, dairy products, meat, and glucose (in gram per day), as well as daily caloric intake (kcal/day) were also collected during the dietary questionnaire.

### 2.5. Statistical Analysis

Baseline was the date of the dietary questionnaire, and participants contributed person-time until the date of RA diagnosis, the last completed questionnaire, death, or loss to follow-up, whichever occurred first.

To estimate the risk of RA associated with fish, lean fish, and oily fish consumption (hazard ratios (HR) and their 95% confidence intervals (95% CI)), we used Cox multivariable regression models. Model 1 used age as the time scale and was also adjusted on daily caloric intake. Model 2 was further adjusted on education level, BMI and physical activity, personal cigarette smoking, exposure to passive smoking in childhood, and gastrointestinal transit, which have been shown to be associated with RA in our cohort [20].

Because smoking plays a role in RA physiopathology, we assessed a potential interaction between smoking and fish consumption. We also stratified the analyses based on the smoking status (smokers (current or former) and non-smokers).

## 3. Results

### 3.1. Study Population

**Figure 1:**
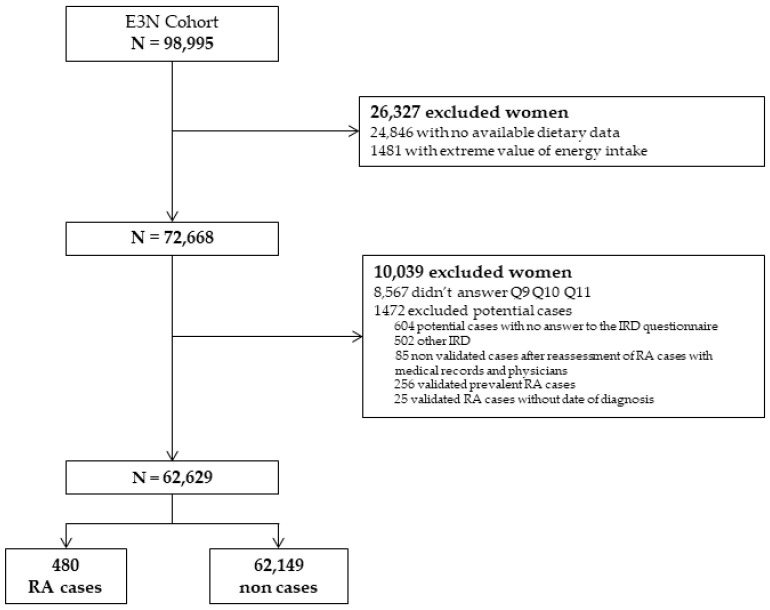
Flow chart of the study. RA: rheumatoid arthritis; IRD: inflammatory rheumatic disease; E3N: Etude Epidémiologique auprès des femmes de la Mutuelle générale de l’Education Nationale.

Among the 98,995 women of the cohort, the dietary questionnaire was completed by 72,668 participants. Among them, 62,639 women met the inclusion criteria, and 480 incident RA cases were detected (Figure 1). The mean (SD) time between baseline and RA diagnosis was 11.7 (±5.8) years, and the mean age at RA diagnosis was 65.2 (±8.3) years.

Among them, 153 (31.9%) cases had positive RF and/or ACPA and were considered seropositive. The seropositive status of the other cases was considered as unknown. 

Participants’ characteristics according to the tertiles of fish consumption are presented in Table 1. Tertile 1 corresponded of a daily fish consumption of <16.7 g/day, tertile 2 corresponded to 16.7 to 31.1 g/day (117 to 218 g/week or approximately 1 to 2 servings/week), tertile 3 corresponded to >31.1 g/day. Only 1356 women (1.9%) had no consumption of lean and/or oily fish. The mean (SD) consumption of lean fish was 14.0 (10.7) g/day, and the mean (SD) consumption of oily fish was 14.4 (9.32) g/day. The mean (SD) daily caloric intake was 2136 (542.4) kcal per day.

### 3.2. Overall Fish Consumption and Risk of RA

Associations between tertiles of overall fish consumption and the risk of RA are presented in Table 2. Compared with low fish consumption (tertile 1; ≤16.7 g/day), a moderate consumption (tertile 2) was associated with a lower risk of RA (HR 0.74; 95% CI [0.59; 0.94] in model 2). However, no association was found with a high consumption of fish (tertile 3) (HR 0.99; 95% CI [0.80; 1.22] in model 2). Overall, there were no linear association with fish consumption (*p* for linear trend 0.65).

We did not find any interaction between fish consumption and smoking (*p* for interaction 0.80). However, as prespecified, we conducted stratified our analyses based on personal cigarette smoking (smokers (current or former) or non-smokers). Among smoking women (current or former), but not among non-smoking women, a moderate consumption of fish (tertile 2) was associated with a decreased risk of RA (HR 0.61; 95% CI 0.44–0.85 in model 2).

### 3.3. Lean Fish and Risk of RA

Associations between tertiles of lean fish consumption, including hake, pollack, common ling, dab, haddock, sole, whiting, and/or cod, and incident RA are presented in Table 3. In the whole population, as well as in stratified analyses on personal smoking status, we did not find any association between lean fish consumption and the risk of RA (HR 1.03; 95% CI [0.83; 1.28] in model 2) for a moderate consumption of lean fish compared to a low consumption.

### 3.4. Oily Fish and Risk of RA

Associations between tertiles of oily fish consumption, including sardine, mackerel, salmon, trout, and/or tuna, and incident RA are presented in Table 4. In the overall population, we found a trend toward a reduced risk of RA with moderate fish oily fish consumption, although it was not statistically significant (HR 0.81; 95% CI [0.65; 1.01]).

There was no interaction between oily fish consumption and the risk of RA (*p* for interaction = 0.25) However, as prespecified, we conducted stratified our analyses based on personal cigarette smoking. We found an inverse association between moderate fish consumption and the risk of RA among non-smokers (HR 0.70; 95% CI [0.51; 0.98] in model 2), but not among smoking women (current or former).

## 4. Discussion

In our large population-based cohort study of French women, there was a U-shaped relationship between overall fish consumption and the risk of RA, with a reduced RA risk only in moderate consumers (tertile 2 roughly corresponding to one to two weekly servings of fish). The inverse association was restricted to current of former smokers. When separately considering oily and lean fish, the second tertile of oily fish consumption was inversely associated with the risk of RA only in non-smoking women, while there was no association whatsoever with lean fish.

A non-linear association between fish consumption and RA risk was previously reported in a non-linear approach for modelling by Di Giuseppe et al. [5]. In their dose-response meta-analysis including seven studies (four case-control studies and three prospective cohort studies), when fish consumption was modelled using restricted cubic splines, the risk of RA was decreased for up to two servings per week, followed by a slight increase in risk for higher consumption. Numerous studies support the potential benefits of omega-3 fatty acids, mainly docosahexaenoic (DHA) and eicosapentaenoic acid (EPA), on metabolic diseases based on their antioxidant and anti-inflammatory properties [21]. A potential explanation by the authors might be a balance of omega-3 fatty acids with the presence of contaminants such as polychlorinated biphenyls, which have been found to be positively associated with RA [22]. Thus, an excess of fish consumption and of those contaminants could counterbalance the potential protective effects of omega-3 fatty acids.

The inverse association between moderate overall fish consumption and the risk of RA seemed restricted to smokers (current or former). Similar results were reported in the Nurses’ Health study I and II, where fish intake attenuated the strong association with smoking for RA diagnosed ≤55 years of age, suggesting that fish intake may exert effects by lowering inflammation due to smoking [8]. The authors found in their cohort an interaction between fish consumption and smoking. In another study of our group, we reported an inverse association between adherence to the Mediterranean diet and RA risk among current or former smokers but not among non-smokers, which is consistent with the present findings and suggests some benefit against the tobacco-associated risk [23]. Thus, we hypothesised that those findings could be explained by the differences in RA pathophysiologic mechanisms between smokers and non-smokers. Increased oxidant effect of smoking might be counterbalanced by the antioxidant effect of the Mediterranean diet and/or omega-3 fatty acids intake. On the other hand, the potential benefit of omega-3 fatty acids intake could be lost by the increased intake of contaminants, especially mercury and endocrine disruptors, with a high consumption of fish.

Finally, we found a trend towards an inverse association between moderate consumption of oily fish, rich in omega-3 fatty acids, and the risk of RA, but not with lean fish. Those results are consistent with a previous study of Pederson et al., using the Danish National Patient Registry, including 57,053 participants and 69 incident RA cases [11]. They separately analysed the association between lean fish and oily fish with the risk of RA. Although not statistically significant, relative risks were 0.62 (95% CI [0.32; 1.22]) with oily fish and 0.83 (95% CI [0.47; 1.46]) for lean fish, respectively. In our study, an inverse association was only found among non-smokers. Even if these results are different from those found with total fish consumption, they suggest that a high portion of omega-3 fatty acids might have a protective role on autoimmunity itself, independently of smoking. Those results seem to be confirmed in a recently published VITAL randomised controlled trial, investigating the benefit of vitamin D and marine-derived omega-3 fatty acids to reduce autoimmune disease risk among 25,871 participants followed for a median of 5.3 years [24]. Although not statically significant, probably due to a lack of power, there was a trend toward a reduced risk of confirmed or probable RA with omega-3 supplementation (HR 0.57; 95% CI [0.31; 1.05]; *p* = 0.07). To our knowledge, it was the first time that this risk was assessed within a randomised controlled trial, and the long-term results will be of interest.

We acknowledge some limitations to our study. First, our cohort only included French women, but with RA being more frequent among women, our study population was appropriate. Nevertheless, our population mainly included teachers, whose dietary habits could differ from the general French population. In addition, identification of RA cases was based on self-reported questionnaire, but we improved the accuracy of our case identification by using a specific questionnaire and a medication database. Finally, fish consumption was assessed only at a single time, and women could have changed their dietary habits, and some associations had moderate effect size, which could lead to cautious interpretations of our findings.

Nevertheless, our study has several strengths including the cohort size, the number of RA cases, and the long follow-up period. In addition, we were able to separately analyse lean fish consumption and oily fish consumption, and the trend toward a reduced risk of RA was only found with oily fish consumption, which is rich in omega-3 fatty acids.

## 5. Conclusions

In conclusion, while there is no strong linear association between fish consumption and the risk of RA, moderate fish consumption, roughly corresponding to one to two weekly portions of fish, could reduce this risk and potentially counterbalance the increased risk of RA induced by smoking. Further studies on larger datasets are needed to further investigate the potential effects of oily and lean fish on RA risk.

## Figures and Tables

**Table 1:** Characteristics of the study population at baseline by tertiles of fish consumption (N = 62,629).

		Fish Consumption		
	All (N = 62,629)	1st Tertile (0–16.7 g/day) (N = 20,681)	2nd Tertile (16.7–31.1 g/day) (N = 19,749)	3rd Tertile (31.1–261 g/day) (N = 22,199)
Age at baseline (years)	52.5 (6.5)	52.3 (6.5)	52.1 (6.4)	53.1 (6.5)
Educational level				
<High School	8322 (13.3)	3091 (14.9)	2542 (12.8)	2689 (12.1)
Up to two-level university	32,032 (51.1)	10,754 (52.0)	10,123 (51.3)	11,155 (50.3)
≥Three-level university	22,275 (35.6)	6836 (33.1)	7084 (35.9)	8355 (37.6)
Smoking status				
Current smoker	8269 (13.2)	2779 (13.5)	2532 (12.8)	2958 (13.3)
Non smoker	33,558 (53.6)	11,401 (55.1)	10,622 (53.8)	11,535 (52.0)
Former smoker	20,802 (33.2)	6501 (31.4)	6595 (33.4)	7706 (34.7)
Passive smoking in childhood	8972 (14.3)	3043 (14.7)	2880 (14.6)	3049 (13.7)
Gastrointestinal transit				
Normal	45,104 (72.0)	14,814 (71.6)	14,319 (72.5)	15,971 (71.9)
Diarrhoea	1720 (2.8)	569 (2.8)	541 (2.8)	610 (2.8)
Constipation	8579 (13.7)	2922 (14.1)	2631 (13.3)	3026 (13.6)
Alternating diarrhoea/constipation	7226 (11.5)	2376 (11.5)	2258 (11.4)	2592 (11.7)
Body mass index (kg/m^2^)	22.9 (3.2)	22.7 (3.1)	22.8 (3.2)	23.2 (3.3)
Physical activity (MET.h/week)	44.8 (28.7)	44.2 (28.7)	44.2 (28.1)	45.9 (29.4)
Hypothyroidism	1287 (2.1)	366 (1.8)	401 (2.0)	520 (2.4)
Diabetes mellitus	3780 (6.0)	1106 (5.3)	1087 (5.5)	1587 (7.1)
Daily caloric intake (kcal/day)	2136.3 (542.4)	2034.9 (525.4)	2159.0 (529.4)	2210.4 (555.1)
Food consumption				
Fish (g/day)	28.6 (21.7)	9.6 (4.3)	23.0 (3.5)	51.3 (20.6)
Fruits (g/d day)	255.0 (169.2)	244.7 (171.2)	248.7 (161.8)	270.3 (172.6)
Vegetables (g/day)	278.4 (134.6)	245.4 (125.6)	271.0 (124.2)	315.7 (142.3)
Olive oil (g/day)	4.8 (5.6)	3.8 (5.1)	4.5 (5.3)	5.8 (6.2)
Cereal (g/day)	191.2 (99.2)	186.6 (98.0)	194.0 (97.2)	193.1 (102.0)
Dairy products (g/day)	249.7 (194.3)	235.3 (190.7)	243.3 (187.2)	268.8 (202.3)
Meat (g/day)	103.4 (50.2)	97.4 (50.4)	109.3 (50.4)	103.7 (49.1)
Glucose (g/day)	236.1 (61.8)	230.9 (61.3)	238.6 (61.2)	238.7 (62.4)
Incident RA after baseline	480 (0.8)	172 (0.8)	121 (0.6)	187 (0.8)

Results are expressed as N (%) for categorical variables and mean (STD) for continuous variables. Abbreviations: RA: rheumatoid arthritis; MET: metabolic equivalents of task: g: gram; kcal: kilocalories.

**Table 2:** Hazard ratios (95% confidence intervals) for the risk of rheumatoid arthritis (RA) by tertiles of fish consumption (N = 62,629).

Fish Consumption	Non-Cases N (%)	RA N (%)	Model 1 HR (95% CI)	Model 2 HR (95% CI)
**All population**	N = 62,149	N = 480		
Tertile 1 (0–16.7 g/day)	20,509 (33.00)	172 (35.83)	Reference	Reference
Tertile 2 (16.7–31.1 g/day)	19,628 (31.58)	121 (25.21)	0.74 [0.58; 0.93]; *p* = 0.01	0.74 [0.59; 0.94]; *p* = 0.01
Tertile 3 (31.1–261 g/day)	22,012 (35.42)	187 (38.96)	0.99 [0.80; 1.22]; *p* = 0.99	0.99 [0.80; 1.22]; *p* = 0.92
*p*_trend_			0.63	0.65
**Non-smokers**	N = 33,314	N = 244		
Tertile 1 (0–16.7 g/day)	11,322 (33.99)	79 (32.38)	Reference	Reference
Tertile 2 (16.7–31.1 g/day)	10,556 (31.69)	66 (27.05)	0.89 [0.64; 1.24]; *p* = 0.48	0.90 [0.65; 1.25]; *p* = 0.53
Tertile 3 (31.1–261 g/day)	11,436 (34.33)	99 (40.57)	1.19 [0.88; 1.60]; *p* = 0.25	1.21 [0.90; 1.64]; *p* = 0.21
*p*_trend_			0.15	0.12
**Smokers (current or former)**	N = 28,835	N = 236		
Tertile 1 (0–16.7 g/day)	9187 (31.86)	93 (39.41)	Reference	Reference
Tertile 2 (16.7–31.1 g/day)	9072 (31.46)	55 (23.31)	0.60 [0.43; 0.84]; *p* = 0.003	0.61 [0.44;0.85]; *p* = 0.003
Tertile 3 (31.1–261 g/day)	10,576 (36.68)	88 (37.29)	0.81 [0.60; 1.09]; *p* = 0.16	0.81 [0.60; 1.08]; *p* = 0.14
*p*_trend_			0.39	0.36

M1: Adjusted for age (as the time scale) and total daily intake except alcohol (kcal/d). M2: M1 and adjusted on body mass index (kg/m^2^) (<18.5 kg/m^2^, 18.5–25 kg/m^2^, 25–30 kg/m^2^, ≥30 kg/m^2^), cigarette smoking (current smoker, former smoker, or non-smoker, except for stratified analyses), exposure to passive smoking in childhood (no, yes), gastrointestinal transit (normal, diarrhoea, constipation, alternating diarrhoea/constipation), educational level (<high school, up to two-level university, three-level university or more), and physical activity (in quartiles of metabolic equivalents of task per week).

**Table 3:** Hazard ratios (95% confidence intervals) for the risk of rheumatoid arthritis (RA) by tertiles of lean fish consumption, including hake, pollack, common ling, dab, haddock, sole, whiting, and/or cod (N = 62,629).

Daily Lean Fish Consumption	Non-Cases N (%)	RA N (%)	Model 1 HR (95% CI)	Model 2 HR (95% CI)
**All population**	N = 62,149	N = 480		
Tertile 1 (0–5.14 g/day)	20750 (33.39)	159 (33.13)	Reference	Reference
Tertile 2 (5.14–16.7 g/day)	20701 (33.31)	164 (34.17)	1.01 [0.82; 1.26]; *p* = 0.89	1.03 [0.83; 1.28]; *p* = 0.80
Tertile 3 (16.7–181.8 g/day)	20698 (33.30)	157 (32.71)	0.95 [0.76; 1.18]; *p* = 0.64	0.96 [0.77; 1.20]; *p* = 0.71
*p*_trend_			0.6104	0.6708
**Non-smokers**	N = 33,314	N = 244		
Tertile 1 (0–5.14 g/day)	11063 (33.21)	77 (31.56)	Reference	Reference
Tertile 2 (5.14–16.7 g/day)	11291 (33.89)	85 (34.84)	1.06 [0.78; 1.45]; *p* = 0.70	1.07 [0.79; 1.46]; *p* = 0.65
Tertile 3 (16.7–181.8 g/day)	10960 (32.90)	82 (33.61)	1.02 [0.75; 1.39]; *p* = 0.91	1.03 [0.76; 1.41]; *p* = 0.84
*p*_trend_			0.9455	0.8800
**Smokers (current or former)**	N = 28,835	N = 236		
Tertile 1 (0–5.14 g/day)	9687 (33.59)	82 (34.75)	Reference	Reference
Tertile 2 (5.14–16.7 g/day)	9410 (32.63)	79 (33.47)	0.97 [0.71; 1.33]; *p* = 0.87	0.99 [0.73; 1.35]; *p* = 0.97
Tertile 3 (16.7–181.8 g/day)	9738 (33.77)	75 (31.78)	0.88 [0.64; 1.21]; *p* = 0.43	0.89 [0.65; 1.22]; *p* = 0.46
*p*_trend_			0.4171	0.4413

M1: Adjusted for age (as the time scale) and total daily intake except alcohol (kcal/d). M2: M1 and adjusted on body mass index (kg/m^2^) (<18.5 kg/m^2^, 18.5–25 kg/m^2^, 25–30 kg/m^2^, ≥30 kg/m^2^), cigarette smoking (current smoker, former smoker, or non-smoker, except for stratified analyses), exposure to passive smoking in childhood (no, yes), gastrointestinal transit (normal, diarrhoea, constipation, alternating diarrhoea/constipation), educational level (<high school, up to two-level university, three-level university or more), and physical activity (in quartiles of metabolic equivalents of task per week).

**Table 4:** Hazard ratios (95% confidence intervals) for the risk of rheumatoid arthritis (RA) by tertiles of oily fish consumption including sardine, mackerel, salmon, trout, and/or tuna (N = 62,629).

Daily Oily Fish Consumption	Non-Cases N (%)	RA N (%)	Model 1 HR (95% CI)	Model 2 HR (95% CI)
**All population**	N = 62,149	N = 480		
Tertile 1 (0–5.7 g/day)	20,794 (33.46)	171 (35.63)	Reference	Reference
Tertile 2 (5.7–14.3 g/day)	20,736 (33.36)	138 (28.75)	0.81 [0.65; 1.01]; *p* = 0.06	0.81 [0.65; 1.02]; *p* = 0.07
Tertile 3 (14.3–261 g/day)	20,619 (33.18)	171 (35.63)	1.01 [0.81; 1.25]; *p* = 0.95	1.01 [0.81; 1.25]; *p* = 0.96
*p*_trend_			0.5766	0.5868
**Non-smokers**	N = 33,314	N = 244		
Tertile 1 (0–5.7 g/day)	11,570 (34.73)	90 (36.89)	Reference	Reference
Tertile 2 (5.7–14.3 g/day)	11,066 (33.22)	61 (25.00)	0.70 [0.51; 0.98]; *p* = 0.03	0.71 [0.51; 0.98]; *p* = 0.04
Tertile 3 (14.3–261 g/day)	10,678 (32.05)	93 (38.11)	1.10 [0.82; 1.48]; *p* = 0.51	1.12 [0.84; 1.50]; *p* = 0.45
*p*_trend_			0.2026	0.1732
**Smokers (current or former)**	N = 28,835	N = 236		
Tertile 1 (0–5.7 g/day)	9224 (31.99)	81 (34.32)	Reference	Reference
Tertile 2 (5.7–14.3 g/day)	9670 (33.54)	77 (32.63)	0.91 [0.66; 1.24]; *p* = 0.55	0.91 [0.67; 1.24]; *p* = 0.55
Tertile 3 (14.3–261 g/day)	9941 (34.48)	78 (33.05)	0.90 [0.66; 1.23]; *p* = 0.50	0.88 [0.65; 1.21]; *p* = 0.44
*p*_trend_			0.5575	0.4887

M1: Adjusted for age (as the time scale) and total daily intake except alcohol (kcal/day). M2: M1 and adjusted on body mass index (kg/m^2^) (<18.5 kg/m^2^, 18.5–25 kg/m^2^, 25–30 kg/m^2^, ≥30 kg/m^2^), cigarette smoking (current smoker, former smoker, or non-smoker, except for stratified analyses), exposure to passive smoking in childhood (no, yes), gastrointestinal transit (normal, diarrhoea, constipation, alternating diarrhoea/constipation), educational level (<high school, up to two-level university, three-level university or more), and physical activity (in quartiles of metabolic equivalents of task per week).

## Data Availability

Data are available upon reasonable request.
